# MetaboSearch: Tool for Mass-Based Metabolite Identification Using Multiple Databases

**DOI:** 10.1371/journal.pone.0040096

**Published:** 2012-06-29

**Authors:** Bin Zhou, Jinlian Wang, Habtom W. Ressom

**Affiliations:** Department of Oncology, Georgetown University, Washington, DC, United States of America; Aberystwyth University, United Kingdom

## Abstract

Searching metabolites against databases according to their masses is often the first step in metabolite identification for a mass spectrometry-based untargeted metabolomics study. Major metabolite databases include Human Metabolome DataBase (HMDB), Madison Metabolomics Consortium Database (MMCD), Metlin, and LIPID MAPS. Since each one of these databases covers only a fraction of the metabolome, integration of the search results from these databases is expected to yield a more comprehensive coverage. However, the manual combination of multiple search results is generally difficult when identification of hundreds of metabolites is desired. We have implemented a web-based software tool that enables simultaneous mass-based search against the four major databases, and the integration of the results. In addition, more complete chemical identifier information for the metabolites is retrieved by cross-referencing multiple databases. The search results are merged based on IUPAC International Chemical Identifier (InChI) keys. Besides a simple list of m/z values, the software can accept the ion annotation information as input for enhanced metabolite identification. The performance of the software is demonstrated on mass spectrometry data acquired in both positive and negative ionization modes. Compared with search results from individual databases, MetaboSearch provides better coverage of the metabolome and more complete chemical identifier information. Availability: The software tool is available at http://omics.georgetown.edu/MetaboSearch.html.

## Introduction

Metabolite identification is one of the key challenges in current mass spectrometry-based untargeted metabolomics studies. At present, metabolite identification is mainly achieved through mass-based search followed by verification using authentic compounds. First, the mass-to-charge ratio (m/z) of a molecular ion of interest is searched against metabolite database(s). The molecules having molecular weights within a specified tolerance to the query m/z value are retrieved from the databases as putative identifications. Then, authentic compounds of these putative identifications are subjected to a tandem mass spectrometry experiment side-by-side with the sample. By comparing the MS/MS spectra and retention times of the authentic compounds with the molecules of interest in the sample, the identities of the molecules are confirmed.

Although the mass-based search alone is inadequate to identify a metabolite, it is an important step in the identification process since it provides putative candidates for subsequent confirmation. Multiple metabolite databases have been assembled during the past years [1], [2], [3], [4]. However, none of the existing databases guarantees a complete coverage of the metabolome. In addition, data formats and retrieval methods are different among databases. As a result, it is desirable to search against multiple databases to increase the metabolome coverage. The search results from multiple databases need to be combined into a non-redundant and uniform format for easy interpretation. Particularly, an efficient and automated computational solution is desired for the identification of a large number of metabolites comparing with the manual retrieval and combination from multiple databases.

Due to the presence of multiple types of derived ions such as sodium/potassium adducts, isotopes, and in-source fragments, ion annotation is utilized to assist in metabolite identification [5]. For example, the open source software CAMERA allows ion annotation based on the similarity between the extracted ion chromatograms of peaks from liquid chromatography-mass spectrometry (LC-MS) data and the m/z difference between these peaks [6]. A tool similar to CAMERA is described in [7]. Through ion annotation, ions originating from the same compound can be recognized and their adduct/isotope/in-source fragment formations can be annotated. Appropriate accurate masses of these ions are calculated based on the annotation information, which in turn improves the accuracy of mass-based search.

In this paper, we introduce a web-based software tool called MetaboSearch that can perform simultaneous search against four metabolite databases: Human Metabolome DataBase (HMDB) [4], Madison Metabolomics Consortium Database (MMCD) [1], Metlin [2], and LIPID MAPS [3]. The search results are then integrated into a non-redundant format. Various types of chemical identifiers are provided by the software for effective reference of metabolites, which consist of PubChem Compound ID (CID), PubChem Substance ID (SID), HMDB ID, KEGG ID, IUPAC International Chemical Identifier (InChI) string, and InChI key. In addition to a simple list of m/z values, the output file from CAMERA can be uploaded into MetaboSearch, so that the ion annotation information on different product ion formations can be used for enhanced mass-based metabolite identification.

MetaboSearch can be used to increase the coverage of metabolome by leveraging the information from multiple databases. Previously, a similar database, MZedDB, was reported in [8]. However, three things distinguish MetaboSearch from MZedDB: (1) MetaboSearch directly retrieves the data from the online databases at each time of mass-based identification. Thus the results of MetaboSearch are derived from the most current information available at the databases, while MZedDB needs to update its database at the sever side every three months; (2) MetaboSearch is intended for batch search, where multiple m/z values can be directly uploaded and searched, while MZedDB needs additional R-code for batch search of multiple m/z values of interest; (3) whereas MZedDB considers different derived ions of metabolites by allowing users to customize the adduct formations used, MetaboSearch can accept the ion annotation information from CAMERA to avoid guessing the possible adduct formations of an ion.

## Methods

### Design

MetaboSearch is designed to accept two types of inputs: a list of m/z values or m/z values along with annotation information from CAMERA. With both types of inputs, the putative identifications of each m/z value are retrieved according to the given mass tolerance. The databases from which the putative identifications are obtained can be selected by users tailoring to their specific needs. For example, in human metabolomics research, the user has the option to search in HMDB only, whose entries are known human metabolites. Alternatively, the user may choose to search in other databases besides HMDB, in which case more putative identifications can be acquired but with the potential risk of getting identifications from other organisms.

**Figure 1:**
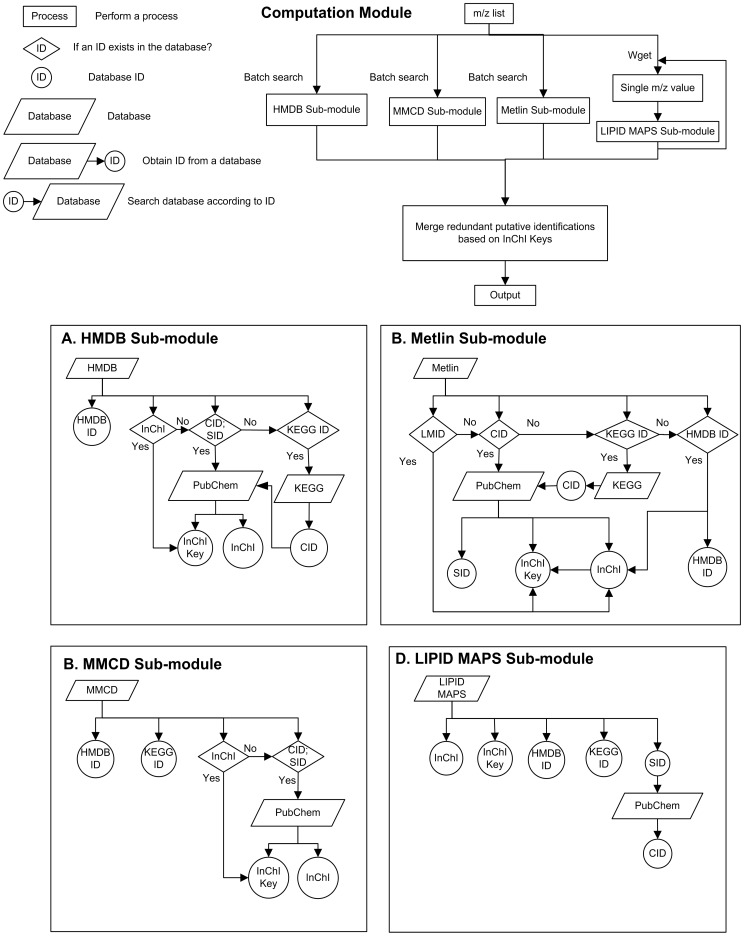
The flowchart of the computation module.

When only a list of m/z values are provided for identification, every m/z value is treated as a (de)protonated ion. First, metabolites from each selected database are retrieved if their masses fall within the mass tolerance range of the input m/z values. A list of chemical identifiers referring to these metabolites are acquired including PubChem CID, PubChem SID, KEGG ID, HMDB ID, InChI string and InChI key. For databases which provide only limited chemical identifier information, the missing identifiers are filled by cross referencing from other databases. For example, in Metlin, neither InChI string nor InChI key is provided. In addition, a large fraction of PubChem CID information is missing. MetaboSearch fills out these missing identifiers by retrieving the missing information from available hyperlinks directing to LIPID MAPS, KEGG or HMDB database. Finally, the search results from individual database are merged through InChI keys. The InChI key is a hashed version of InChI string which describes the atoms and bond connectivity information, tautomeric information, isotope information, stereochemistry information, and electronic charge information of a molecule. It has been previously demonstrated that InChI and InChI key provide the best way to describe chemical compounds through structure codes [9]. InChI key generally consists of 25 characters, where the first 14 characters contain the connectivity information of a molecule. Since the stereochemistry is generally less a concern in the initial identification of a metabolite, and the stereochemistry information from database is sometimes incomplete or missing, we choose to merge the stereoisomers into the same entry in the final search results. By combining compounds having identical first 14 characters of InChI keys, the same metabolites and stereoisomers are merged while structural isomers are kept as different metabolites. The possible number of stereoisomers is presented in the search results by counting the InChI keys with the same first 14 characters but different remaining characters. The flowchart of mass-based search and results integration is shown in Figure 1 as the computation module of the software.

When the output annotation file from CAMERA is imported into the software, the annotation file is first parsed to group ions originating from the same metabolite together. If conflicting annotation is encountered with an ion having multiple possible annotations, the ion is included in multiple annotation groups and identified separately. After parsing, the monoisotopic protonated (or deprotonated in negative ionization mode) m/z of each annotation group is calculated. These m/z values are then used to search against databases, as these m/z values are directly acquired from a list of m/z values only, which is described in the previous paragraph. Following the mass-based identification, the search results are combined so that the ion with multiple annotations is represented as a single ion with putative identifications from multiple annotation groups.

### Implementation

**Figure 2:**
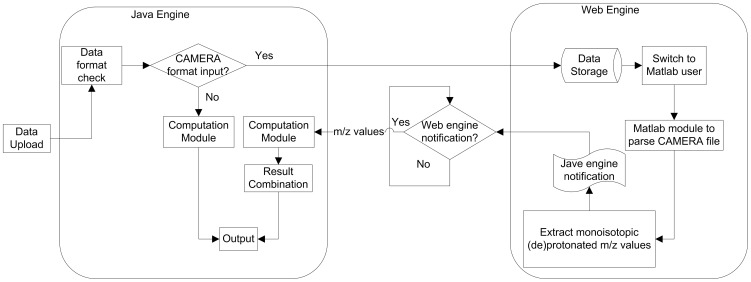
The software architecture of MetaboSearch.

Two techniques can be used to integrate information from multiple databases: a data warehouse or web-based techniques. A data warehouse has a backend database combines the data from the different sources, and provides a unified, reconciled view of architected data environment that serves as the single integrated source of data for information storage and retrieval. Compared with data warehouse, web based techniques require no local storage or management while are able to synchronize the results with their source databases. Since the knowledge base of metabolites is in constant expansion, web based implementation is used for MetaboSearch. Java language is often adopted for web application due to its outstanding performance across platforms. MetaboSearch is implemented using Java and C coupled with web spidering and PHP techniques. The architecture of the software is composed of two parts: a Java engine and a Web engine, as shown in Figure 2. The function of each engine is described below.

**Figure 3:**
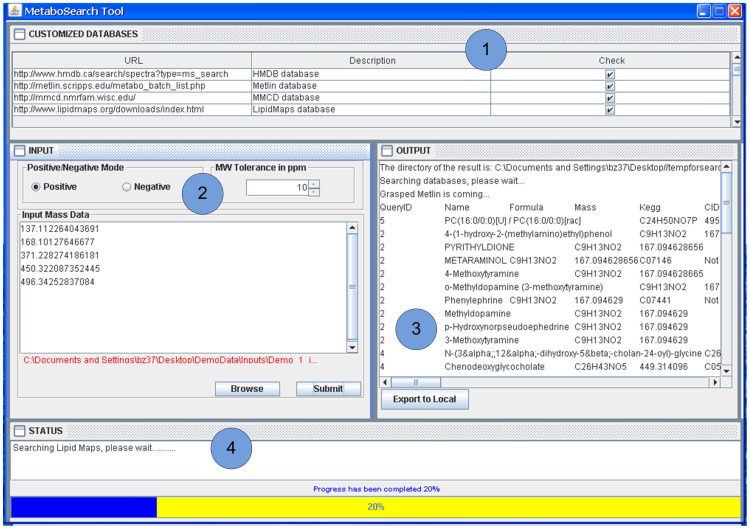
MetaboSearch interface. There are four tabbed panels in the software.

Java engine is responsible for importing data, database search, and exporting results. The computation module depicted in Figure 1 is the major module within the Java engine. In the computation module, each database is searched respectively through a sub-module. The retrieval results are dynamically fetched from online databases through web spidering. For HMDB, MMCD and Metlin, the batch query function from these databases is used. The webpage data of the metabolites are analyzed for information extraction and cross referencing. For LIPID MAPS, open source software GNU Wget is used to query the database. Wget iterates over the m/z value list. For each m/z value subjected to search, metabolite information is downloaded to a local file. Then, the file is parsed to extract the relevant information. Finally, the metabolite lists from multiple databases are integrated to remove the redundant metabolites.

The web engine focuses on parsing the ion annotation information from the input file. The input file is first stored in a temporary storage space on the server after it is imported. Then a shell script calls a Matlab application to parse the file and calculate the monoisotopic (de)protonated m/z values of each peak. During the execution of the web engine, the Java engine constantly queries the status of the web engine. Once the annotation information is parsed, the web engine sends a message to notify the Java engine. The Java engine then conducts the mass-based search for metabolites, but using the calculated monoisotopic (de)protonated m/z values from the web engine. Besides the integration of the database retrieval results, the Java engine is responsible to put the previously split ions (due to conflicting ion annotation) back into a single ion and combine their putative identifications.

To reduce the usage of internet bandwidth and computation load of the web server, MetaboSearch uses the Java Web Start technique, which enables most of the computational task to be executed locally on the users’ machines. Java Web Start resorts to Java Network Launching Protocol (JNLP), so that the web browser can automatically download, cache and run Java software. Since JNLP is cross-platform, MetaboSearch can be run under Windows, Linux or Mac platform.

### Software Interface

MetaboSearch has four tabbed panels as shown in Figure 3. Panel 1 enables the user to select databases to search against. Panel 2 is used to import data, set the ionization mode of data and the mass tolerance for the search. The input data can be either directly pasted on the panel (for m/z values only) or be imported from an Excel (.xls) file (for both m/z values and CAMERA output files). Panel 3 displays the search results from individual databases. The bottom “Export to Local” button outputs the integrated results in Excel (.xls) format. Panel 4 shows the program running status and progress.

## Results and Discussion

Two datasets (Dataset 1 and Dataset 2) were analyzed to showcase the efficacy of MetaboSearch in mass-based metabolite identification. The datasets were acquired by analyzing human serum samples through an ultra performance liquid chromatography coupled with a hybrid quadrupole time-of-flight mass spectrometer (UPLC-QTOF MS) and by preprocessing the raw LC-MS data using the XCMS software [10]. Dataset 1 was first analyzed by CAMERA for ion annotation. Then the output was subjected to mass-based search to acquire putative identifications. Dataset 2 consists of a list of m/z values that was directly imported into MetaboSearch to acquire putative identifications.

In Dataset 1, 724 peaks previously acquired in the positive mode were annotated by CAMERA for four types of adducts ([M+H]^+^, [M+Na]^+^, [M+K]^+^, [M+H-H2O]^+^), isotopes up to 4^th^ order and doubly charged ions. After importing into MetaboSearch, the peaks were assigned to 497 annotation groups. The m/z values of these annotation groups were then used for mass-based identification with a 10 ppm (parts-per-million) mass tolerance. If no ion annotation information is used during the database search, all peaks would be assumed to be monoisotopic, protonated ions. Out of 724 peaks, only 280 peaks would have at least one putative identification compared with the 331 peaks having at least one putative identification when ion annotation is used.

In Dataset 2, a list of 423 m/z peaks previously acquired in the negative mode was fed to MetaboSearch for putative identifications. In this analysis, no annotation information associated with the list was used. Therefore, all peaks were assumed to be monoisotopic deprotonated ions. An m/z tolerance of 10 ppm was used.

**Table 1:** The number of putative identifications acquired by each of the four databases compared to MetaboSearch.

Datasets	HMDB	MMCD	Metlin	LipiMaps	MetaboSearch
Dataset 1	361 (147)	673 (251)	1180 (244)	525 (145)	1819 (331)
Dataset 2	177 (63)	360 (109)	748 (107)	469 (64)	1237 (144)

The figures in parenthesis indicate the number of m/z values with at least one putative identification.

Table 1 summarizes the putative identification results obtained for Dataset 1 and Dataset 2. As shown in the table, MetaboSearch retrieves more putative identifications than any single metabolite database. This is expected as MetaboSearch combines the output from individual databases. It should be noted that there is no guarantee that the gained metabolites are correct identifications. As outlined in [11], at least two independent and orthogonal data (retention time and mass spectrum, accurate mass and tandem mass spectrum, etc.) relative to an authentic compound analyzed under identical experimental conditions are necessary to verify a putative metabolite identification. However, MetaboSearch provides a large candidate identification pool for MS-based metabolomics studies, which reduces the possibility that a correct identification is missed at an early stage from further verification due to database limitations. Incorrect putative identifications can be ruled out at a later stage through retention times or tandem mass spectra. On the other hand, if a correct putative identification is missed during the mass-based search, it will be much harder to recover it during the metabolite verification. MetaboSearch also offers information about the database from which a particular putative identification is from. This information may also help users to prioritize the putative identifications as users may have particular preference/confidence on some databases while other databases can be used as reference when the preferred databases do not give satisfactory results.

**Table 2:** Comparison of individual databases with MetaboSearch for the same metabolite on the provided chemical identifiers.

Database	Common Name	KEGG ID	PubChem CID	PubChem SID	HMDB ID
MetaboSearch	(3a,5b,7b)-24-[(carboxymethyl)amino]-7-hydroxy-24-oxocholan-3-yl-b-D-glucopyranosiduronic acid; Glycochenodeoxycholic acid 3-glucuronide	C03033	44263370	85300921	HMDB02618;HMDB02579
Metlin	Glycochenodeoxycholic acid 3-glucuronide; (3a,5b,7b)-24-[(carboxymethyl)amino]-7-hydroxy-24-oxocholan-3-yl-b-D-glucopyranosiduronic acid				HMDB02618;HMDB02579
LIPID MAPS	Glycochenodeoxycholic acid 3-glucuronide			85300921	HMDB02579
HMDB	Glycochenodeoxycholic acid 3-glucuronide	C03033			HMDB02579
MMCD	Glycochenodeoxycholic acid 3-glucuronide; (3a,5b,7b)-24-[(carboxymethyl)amino]-7-hydroxy-24-oxocholan-3-yl-b-D-glucopyranosiduronic acid				HMDB02618;HMDB02579|

In addition to a more complete coverage of possible metabolites, MetaboSearch also provides more comprehensive information concerning chemical identifiers which are used to refer to a specific metabolite. An example is shown in Table 2, where a negative ion [M-H]^-^ with m/z value 624.3401 was subjected to mass-based identification. Although the ion can be found in all four databases, MetaboSearch provides the most comprehensive chemical identifier information about this metabolite.

MetaboSearch took about 3 hours to acquire putative identifications for Dataset 1 and about 3.5 hours for Dataset 2, on a PC with Quad 2.83 GHz CPU and 8 GB memory running a Windows XP 64-bit operating system. The running time generally depends on the number of peaks/metabolites to be identified and the network condition while searching. Considering the difficulty and the required effort to search and integrate putative identifications manually, we believe this running time is acceptable, especially when the identification of hundreds to a few thousand ions is desired.

### Availability and Future Directions

As a web-based software tool, MetaboSearch is developed to search multiple metabolite databases simultaneously according to the m/z values of observed peaks and output of CAMERA. The search results from individual databases are combined through InChI keys. More complete chemical identifier information is provided by cross-referencing. Annotation information from CAMERA can be incorporated for enhanced metabolite identification. MetaboSearch is shown to provide a more comprehensive list of putative identification and better identification coverage of observed peaks in a LC-MS-based metabolomics study. The software and the datasets presented in this manuscript are available at http://omics.georgetown.edu/MetaboSearch.html.

Further expansions of MetaboSearch include adapting the software to more flexible ion annotation file format (for example, the file format used in [7]). More databases can be added to the software to expand the knowledge-base for mass-based metabolite search. Last but not the least, organism specific information of metabolites can be further integrated into the software through techniques such as literature mining to give users more specific information about the organism they investigate.
